# Absence of physiological Ca^2+^ transients is an initial trigger for mitochondrial dysfunction in skeletal muscle following denervation

**DOI:** 10.1186/s13395-017-0123-0

**Published:** 2017-04-10

**Authors:** Chehade Karam, Jianxun Yi, Yajuan Xiao, Kamal Dhakal, Lin Zhang, Xuejun Li, Carlo Manno, Jiejia Xu, Kaitao Li, Heping Cheng, Jianjie Ma, Jingsong Zhou

**Affiliations:** 1 Rush University School of Medicine, Chicago, IL USA; 2 Kansas City University of Medicine and Bioscience, 1750 Independence Ave, Kansas City, MO 64106 USA; 3 Institute of Molecular Medicine, Peking University, Beijing, China; 4 Wexner Medical Center, The Ohio State University, 460 West 12th Avenue, Columbus, OH USA

**Keywords:** E-C coupling, Calcium imaging, Calcium signaling, Calcium intracellular release, Denervation, Mitochondria

## Abstract

**Background:**

Motor neurons control muscle contraction by initiating action potentials in muscle. Denervation of muscle from motor neurons leads to muscle atrophy, which is linked to mitochondrial dysfunction. It is known that denervation promotes mitochondrial reactive oxygen species (ROS) production in muscle, whereas the initial cause of mitochondrial ROS production in denervated muscle remains elusive. Since denervation isolates muscle from motor neurons and deprives it from any electric stimulation, no action potentials are initiated, and therefore, no physiological Ca^2+^ transients are generated inside denervated muscle fibers. We tested whether loss of physiological Ca^2+^ transients is an initial cause leading to mitochondrial dysfunction in denervated skeletal muscle.

**Methods:**

A transgenic mouse model expressing a mitochondrial targeted biosensor (mt-cpYFP) allowed a real-time measurement of the ROS-related mitochondrial metabolic function following denervation, termed “mitoflash.” Using live cell imaging, electrophysiological, pharmacological, and biochemical studies, we examined a potential molecular mechanism that initiates ROS-related mitochondrial dysfunction following denervation.

**Results:**

We found that muscle fibers showed a fourfold increase in mitoflash activity 24 h after denervation. The denervation-induced mitoflash activity was likely associated with an increased activity of mitochondrial permeability transition pore (mPTP), as the mitoflash activity was attenuated by application of cyclosporine A. Electrical stimulation rapidly reduced mitoflash activity in both sham and denervated muscle fibers. We further demonstrated that the Ca^2+^ level inside mitochondria follows the time course of the cytosolic Ca^2+^ transient and that inhibition of mitochondrial Ca^2+^ uptake by Ru360 blocks the effect of electric stimulation on mitoflash activity.

**Conclusions:**

The loss of cytosolic Ca^2+^ transients due to denervation results in the downstream absence of mitochondrial Ca^2+^ uptake. Our studies suggest that this could be an initial trigger for enhanced mPTP-related mitochondrial ROS generation in skeletal muscle.

**Electronic supplementary material:**

The online version of this article (doi:10.1186/s13395-017-0123-0) contains supplementary material, which is available to authorized users.

## Background

Skeletal muscle is responsible for voluntary movements of the entire body. Because it comprises around 40% of whole-body lean mass of a human, skeletal muscle is also essential for maintaining the homeostasis of the whole-body metabolism [1]. Skeletal muscle contraction is under the control of motor neurons. In some disease states, the interaction between motor neuron and skeletal muscle is lost, leading to paralysis and muscle atrophy [2]. Muscle atrophy is defined as a decrease in the muscle cell size and the imbalance between protein synthesis and degradation. The molecular mechanisms underlying protein metabolism in muscle atrophy have been extensively evaluated [3–8]. It is believed that skeletal muscle atrophy is caused by the disturbance of signaling networks, in which mitochondria may play a major role. In fact, mitochondria occupy about 10–15% of the muscle fiber volume [9]. They are not only essential for energy supply but also determine the survival or death of muscle fibers. It has been shown that denervation of skeletal muscle induces a dramatic increase in mitochondrial ROS production [10]. However, the initial cause of the mitochondrial ROS production in denervated skeletal muscle remains elusive [11].

Muscle cells use Ca^2+^ as a messenger to control events ranging from activation of contraction to cell death. Defective intracellular Ca^2+^ signaling has been linked to skeletal muscle dysfunction during aging [12, 13] and in muscular dystrophy (mdx) [14–17]. In non-muscle cells, mitochondria dynamically transport Ca^2+^ and modify its flux into the endoplasmic reticulum, nucleus, and across the plasma membrane to such an extent that they have been named “the hub of cellular Ca^2+^ signaling” [18]. There is strong evidence that mitochondria may have a similar role in skeletal muscle. We previously demonstrated that mitochondria take up Ca^2+^ during excitation-contraction (E-C) coupling following rapid calcium transients in skeletal muscle [19]. We also established that malfunction of this mechanism contributes to neuromuscular degeneration in amyotrophic lateral sclerosis [20]. Mitochondrial Ca^2+^ uptake is believed to help regulate mitochondrial metabolism and ATP synthesis, so that the energy demands of muscle contraction are met [21]. However, Ca^2+^ overload in mitochondria is also a pathological stimulus of ROS generation [22]. It has been shown that prolonged muscle denervation leads to an increased resting cytosolic free Ca^2+^ level, that in turn overloads mitochondria, stimulating ROS production [23, 24]. Following denervation, no action potential can be initiated; therefore, physiological Ca^2+^ transient is lost in the denervated muscle fibers. One essential question to ask is how mitochondria respond to the cessation of physiological Ca^2+^ transients. While published studies mainly focused on the effect of a steady state level of intracellular Ca^2+^ on mitochondrial function, it is not known whether the dynamic change of Ca^2+^ level inside mitochondria in response to the intracellular Ca^2+^ transients is also a player in regulating mitochondrial function.

In this study, we used a transgenic mouse model carrying a mitochondrial biosensor mt-cpYFP, which produces mitoflash signal as a functional indication of ROS-related mitochondrial metabolic function [25, 26]. This model allows us to detect the early changes of ROS-related mitochondrial metabolic function in live skeletal muscle in response to the denervation process. Under voltage-clamp condition, we found that the loss of physiological Ca^2+^ transients or mitochondrial Ca^2+^ uptake could be an initial trigger for mitochondrial dysfunction with increased mitochondrial ROS production in skeletal muscle fibers following denervation.

## Methods

### Generation of transgenic mice

The plasmid (mt-cpYFP/pUCCAGGS) used to generate this transgenic line (mt-cpYFP) is the same as the one developed by Dr. Heping Cheng’s Laboratory to generate the original mt-cpYFP transgenic mice [26, 27]. The genetic background for this transgenic mouse model is B6SJL from the Jackson Laboratory. The mt-cpYFP mice at the age of 2.5–3 months were used. All experiments were carried out in strict accordance with the recommendations in the Guide for the Care and Use of Laboratory Animals of the National Institutes of Health. Protocols on usage of mice were approved by the Institutional Animal Care and Use Committee of Rush University, University of Missouri at Kansas City and Kansas City University of Medicine and Bioscience.

### Muscle denervation procedure

Muscle denervation was performed by transection of the sciatic nerve. During a denervation procedure, the mouse was anesthetized with constant-flow isoflurane inhalation and a small incision was made in the mid-posterolateral area of the thigh, and the sciatic nerve was isolated. In one hind limb, the sciatic nerve was severed and a ~5 mm section was removed. The ends of the nerve were sutured to prevent nerve regrowth. For control experiments (sham), the sciatic nerve was exposed in the contralateral hind limb without being severed. The incisions in both legs were closed again with silk sutures, and the animal was euthanized after 24 h for experiments.

### Isolation of FDB fibers

The animals were euthanized by CO_2_ inhalation followed by cervical dislocation, and the flexor digitorum brevis (FDB) muscles were removed for imaging studies. Individual FDB muscle fibers were isolated using a modified collagenase-digestion method described previously [19]. Briefly, FDB muscles were digested in modified Krebs solution (0 Ca^2+^) containing 0.2% type I collagenase (Sigma), for 35 min at 37 °C. After digestion, muscles were kept in collagenase-free Krebs solution (with Ca^2+^ and 10 mM glucose) at 4 °C and used for imaging within 6 h. All experiments were conducted under the same isolation procedure and the same time window. In addition, both Sham and Denervation procedures were conducted in the same mouse, thus the paired sham and denervated FDB muscles were isolated from the same mouse for each experiment.

### Confocal imaging of mitoflash (cpYFP) signals and image analysis

Zeiss LSM 510 live confocal microscope was used for imaging cpYFP fluorescence. Images were captured with a ×40, 1.2 NA water immersion objective at a sampling rate of 1 s/frame. Dual excitation of mt-cpYFP was achieved by alternating excitation at 405 and 488 nm, and the emission was collected at >505 nm. Experiments were performed at room temperature (23 °C). Time-lapse confocal images were analyzed using custom-developed algorithms written in Interactive Data language (IDL) [28] and ImageJ (NIH). Motion artifacts, background subtraction and photo-bleach correction were taken into account before automatic flash detection, using the built in custom-software.

### Electric stimulation

FDB fibers isolated from denervated and sham-operated mt-cpYFP mice were bathed in Tyrode’s solution. Individual fibers were electrically stimulated with a stimulation protocol described previously with some modifications [29]. Briefly, extracellular platinum wire electrodes were placed in parallel to the cell of interest. A single field stimulation of 350 ms in duration, consisting of 0.5 ms (8–12 V) pulses applied at a frequency of 40 Hz was generated. Fibers that did not visually respond to stimulation were excluded from the analysis. Mitoflash signal was monitored by collecting consecutive *x*−*y* time series confocal images (100 images) immediately before electrical stimulation, 10 and 120 s after termination of the field stimulation.

### Electroporation and gene expression in FDB muscle of adult mice

The procedure was modified from our previous study [20, 30]. The anesthetized mice were injected with 10 μl of 2 mg/ml hyaluronidase dissolved in sterile saline at the ventral side of the hind paws through a 29-gauge needle. One hour later, 5–10 μg of plasmid DNA of pCDNA3/mt11-YC3.6 in 10 μl of sterile saline were injected into the same sites. Fifteen minutes later, two electrodes (gold-plated stainless steel acupuncture needles) were placed at the starting lines of paw and toes, separated by about 9 mm. Twenty pulses of 100 V/cm with 20 ms duration were applied at 1 Hz (ECM 830 Electro Square Porator, BTX Harvard Apparatus). Seven days later, the mice were euthanized and FDB muscles were removed for functional studies.

### Voltage clamp of FDB muscle fibers

The method was modified from our previous study [20, 31]. Muscle fibers expressing mt11-YC3.6 were patched with 0.6–1.0 MΩ pipettes filled with a cesium glutamate-based solution containing 120 mM cesium glutamate, 5 mM EGTA, 10 mM glucose, 10 mM Tris base, 5 mM ATP, 10 mM phosphocreatine, 1 mM Mg^2+^, 100 nM Ca^2+^, and 50 μM M x-rhod-1. An Axopatch 200B amplifier (Axon Instruments, Foster City, CA) was used for whole-cell patch clamp. The fiber was clamped at −80 mV, and changes in cytosolic Ca^2+^ transients were recorded following application of depolarizing voltages. The external solution contained 140 mM triethanolamine CH_3_SO_3_H, 10 mM Hepes, 1 mM CaCl_2_, 3.5 mM MgCl_2_, 1 mM 4-aminopyridine, 0.3 mM LaCl_3_, 0.5 mM CdCl_2_, 0.5 μM tetrodotoxin, and 50 μM *N*-benzyl-*p*-toluenesulfonamide.

### Confocal imaging of YC3.6, x-rhod-1 and MitoSOX Red in FDB muscle fibers

We have developed a method to simultaneously record the images of YC3.6 for mitochondrial Ca^2+^ signaling and x-rhod-1 for cytosolic Ca^2+^ signaling [20]. Separate excitation and emission wavelengths were applied for simultaneous recording of x-rhod-1 and YC3.6 signals. x-rhod-1 was excited at 594 nm, and its fluorescence was collected at 600–680 nm. mt11-YC3.6 was excited at 458 nm, and its fluorescence *f*_1_ was collected at 470–520 nm and *f*_2_ at 520–580 nm. MitoSOX Red (M36008, Invitrogen) was used to evaluate mitochondrial superoxide production level. FDB muscle fibers were incubated with 0.5 μM MitoSOX Red in our modified Krebs solution at 37 °C for 10 min. MitoSOX Red was excited at 514 nm, and its fluorescent images were collected at 570–630 nm under the Leica SP8 confocal microscope.

### Isolation of mitochondrial fraction from skeletal muscles

Crude mitochondrial fractionations from skeletal muscle were first obtained using a method previously reported [32, 33] with some modifications. Briefly, fresh skeletal muscles were removed from the mice and placed in ice-cold phosphate-buffered saline (PBS) with 10 mM EDTA. The muscles were suspended in 10 ml/gram weight ice-cold homogenization buffer (100 mM sucrose, 10 mM EDTA, 100 mM Tris-HCl, 46 mM KCl, pH 7.4 with 5 mg/ml BSA and proteinase inhibitor (Thermo Fisher)), minced into small pieces and homogenized on ice. The homogenate was centrifuged at 800*g* 4 °C for 10 min. The supernatant was transferred to a new centrifuge tube and centrifuged at 10,000*g* 4 °C for 10 min. The resulting pellets were the crude mitochondrial fraction. The supernatant was centrifuged at 100,000*g* 4 °C for 60 min. The final supernatant was the pure cytosolic fraction.

The crude mitochondrial fractions were suspended in 1.5 ml 25% nycodenz buffer, layered onto 1.25 ml 30% nycodenz buffer, and overlaid with 1.25 ml 23% nycodenz buffer containing 5 mM Tris, 3 mM KCl, 0.3 mM EDTA, and pH7.5. The samples were centrifuged at 52,000 for 90 min at 4 °C in a swinging bucket rotor (BECKMAN, SW60 Ti). The mitochondrial fraction was collected from the 25%/30% interface and resuspended in equal volume of homogenization buffer and centrifuged at 10,000*g* at 4 °C for 10 min. This step was repeated for three times. The pure mitochondria fraction in the final pellet was used for the immunoblot assay.

### Immunoblot assay

Protein concentrations were determined by BCA protein assay (Thermo Scientific). Equal mass protein samples (10 *μ*g) were subjected to 10% SDS-polyacrylamide gel electrophoresis, transferred to PVDF membrane (MILIPORE), and immunoblotted with primary antibodies. The antibodies used were anti-Cyclophilin F (Abcam, ab110324), 1:1000 dilution; anti-COX-IV (Cell Signaling, 4844S), 1:5000 dilution and anti-GAPDH (Cell signaling, 5174S), 1:10000 dilution. Results were visualized with ECL reagents (Thermo Scientific). Densitometry evaluation was conducted using ImageJ software (NIH, Bethesda, MD).

### Statistics

Statistical comparisons were done using students *t* test for single mean or ANOVA test for multiple means when appropriate. All graphs were plotted in Sigmaplot (Systat Software Inc.) and results were expressed as mean ± S.E., and *p* < 0.05 was considered significantly different.

## Results

### Denervation leads to drastic increase of mitoflash signal in skeletal muscle fibers

Based on the biochemical study of Muller et al. [10], it has been shown that the ROS production increases 30-fold in skeletal muscle after 7 days of denervation. The initial response of mitochondria to denervation in skeletal muscle has not been studied. The mt-cpYFP transgenic mouse model provides a useful tool to examine the dynamic changes of mitochondrial ROS production in the form of mitoflash signaling in live skeletal muscle cells [25, 26]. While there is ongoing debate regarding the mt-cpYFP sensitivity to ROS and pH [34], resolution of the spatial and temporal aspects of mitoflash signals has been widely used to explore the changes in the metabolic function of mitochondria under physiological and pathological conditions [35].

The mt-cpYFP transgenic mice were generated in our laboratory. The mitochondrial targeting of mt-cpYFP is demonstrated in the Additional file 1: Figure S1. The FDB muscle fibers derived from the mt-cpYFP mice were isolated for live cell imaging to record the mitoflash activity. A standard protocol was established to record the mitoflash events in FDB muscle fibers, in which 100 images were taken continuously at a speed of 1 image/s. The mitoflash signal was analyzed using the established software, FlashSniper [28], to obtain parameters of the mitoflash signal including full area at half maximum (FAHM), full duration at half maximum (FDHM), and the amplitude of the signal (dF/F_0_). In addition, the fiber area giving mitoflash signal during the 100-sequential-imaging time period was summed as Total Flash Area. The ratio of Total Flash Area over the whole fiber area named Total Flash Area/Fiber Area was then calculated. Total Flash Area/Fiber area during this 100-sequential-image recording time period provides quantification of the number of mitochondria in a single muscle fiber that are involved in generating mitoflash events, as well as the frequency of the mitoflash events.

**Fig. 1:**
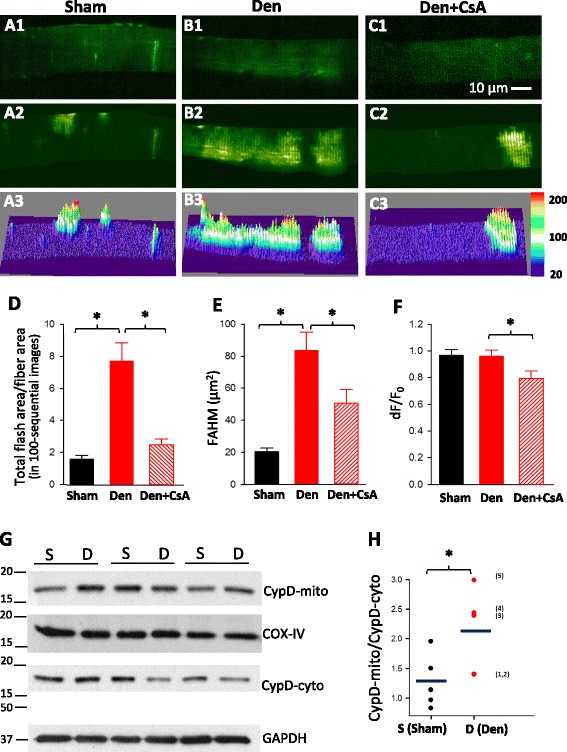
Denervated muscle fibers show an enhanced mitoflash activity, and CypD is involved in this activity. The representative confocal images of muscle fibers isolated from the FDB muscles with sham (**a1–3**), denervation (Den) (**b1–3**), and denervation + CsA treatment (Den + CsA) (**c1–3**). Panels **a1**, **b1**, and **c1** show the initial fluorescence images at *t* = 0 s. Panels **a2, b2**, and **c2** show the peak intensity map of all mitoflashes detected in 100 recorded images. Panels **a3**, **b3**, and **c3** are the 3D surface plots of (**a2**, **b2**, and **c2**). The quantification analysis of the mitoflashes for three conditions are illustrated in **d** *Total Flash Area/Fiber Area* (in 100 sequential images), **e** FAHM, and **F** the amplitude (dF/F_0_) of the mitoflashes. Note that live skeletal muscle fibers respond to the 24-h denervation with dramatically increases of the mitoflash signal in mitochondria, while the CsA treatment restored the Total Flash Area/Fiber Area close to the normal level and significantly reduced the FAHM and signal amplitude (*n* = 57–64, *p < 0.05). **g** Immunoblot assay of CypD expression level in both mitochondria (CypD-mito) and cytosol (CypD-cyto) of the skeletal muscle with and without 24-h denervation. **h** The ratio of CypD in mitochondria over the CypD in cytosol (CypD-mito/CypD-cyto) was quantified. The *annotated numbers in parentheses* indicate the individual immunoblot assay experiments for denervated skeletal muscle. Note that the 24-h denervation promoted the translocation of CypD from cytosol to mitochondria (*n* = 5, *p < 0.05)


To determine the dynamic response of mitochondrial metabolic function of skeletal muscle to denervation, the sciatic nerve transection procedure was conducted at one hind limb of the mt-cpYFP mouse. A sham procedure without sciatic nerve transection was conducted on the contralateral hind limb of the same mt-YFP mouse as the control. To catch the early response of mitochondria to denervation, FDB muscle fibers were isolated for evaluating mitoflash activity 24 h after the procedure. Compared with the sham operation, the denervation resulted in a dramatic increase of mitoflash activity in muscle fibers as shown in the movies (Additional files 2 and 3: Movies 1 and 2) and representative images of a denervated muscle fiber in Fig. 1b1–3. In sham cells, mitoflash events were limited to localized areas of the muscle fiber represented by small patches (Fig. 1a1–3). Upon denervation, there was a significant increase in the fiber area in which mitochondria showed mitoflash signal (Fig. 1b2–3) as quantified by the Total Flash Area/Fiber Area plotted in Fig. 1d (Sham;1.65 ± 0.36, *n* = 64 cells vs Den; 7.77 ± 1.87, *n* = 59 cells; *p* < 0.001). This correlated well with the increase in FAHM (Fig. 1e) of the mitoflash signal (Sham; 20.36 ± 3.6, *n* = 64 muscle fibers vs Den; 83.43 ± 19.14 in μm^2^, *n* = 59 muscle fibers; *p* < 0.001). Despite the dramatic increase in FAHM of the mitoflash signal in denervated muscle fibers, there were no significant changes in other parameters of the mitoflash signal including the amplitude dF/F_0_ (0.97 ± 0.07, *n* = 64 muscle fibers and 0.96 ± 0.09, *n* = 59 muscle fibers; *p* = 0.875) and the FDHM (14.47 ± 2.43, *n* = 58 muscle fibers and 17.84 ± 2.04, *n* = 59 muscle fibers; *p* = 0.08) compared with the sham muscle fibers. These data indicate that in response to denervation, the number of mitochondria producing ROS is increased in the skeletal muscle, while the intensity and the duration of the ROS production of individual mitochondrion remain unchanged.

Additional file 2: movie 1. Mitoflash recording of a denervated muscle fiber; 012Den4231_movie1. (AVI 1458 kb)



Additional file 3: movie 2. Mitoflash recording of a fiber with the sham procedure; 009CL4413_movie2. (AVI 1182 kb)


### Cyclosporine A attenuates the increase of mitoflash activity caused by denervation

It has been shown that mitoflash signal is associated with transient opening of the mitochondrial permeability transition pore (mPTP) in both cardiac and skeletal muscle [26, 27]. However, two published studies reported that CsA did not affect the mitoflash signal in normal skeletal muscle [36, 37], suggesting the possibility that the mitoflash signal is not related to Cyclophilin D (CypD) under physiological conditions. Here, we tested whether mitoflash activity became CypD-dependent upon denervation. We examined whether altered mPTP opening was associated with the generation of massive mitoflash signal in denervated skeletal muscle and whether inhibition of CypD-dependent pore opening could restore mitoflash activity to the normal level in denervated muscles. Muscle fibers isolated from denervated skeletal muscle were treated with 1 μM Cyclosporine A (CsA) for 30 min prior to imaging. Additional file 4 (movie 3) and Fig. 1c1–3 show representative images of denervated muscle treated with CsA. The application of CsA significantly decreased the flashing area (Total Flash Area/Fiber Area) in denervated muscle fibers (Fig. 1d; Den 7.77 ± 1.87, *n* = 59 muscle fibers vs Den + CsA 2.50 ± 0.63, *n* = 57 muscle fibers; *p* < 0.001). In line with those results, the size of mitoflash signal (FAHM) was also significantly reduced (Fig. 1e; Den 83.43 ± 19.14, *n* = 59 muscle fibers vs Den + CsA 50.45 ± 14.96, *n* = 57 muscle fibers, *p* = 0.023). Interestingly, the flash amplitude dF/F_0_ was significantly reduced after the CsA treatment (Fig. 1f; Den 0.96 ± 0.09, *n* = 59 muscle fibers, Den + CsA 0.79 ± 0.10, *n* = 57 muscle fibers; *p* = 0.02). No change in FDHM was observed among the groups. These results indicate that the opening of mPTP plays an important role in the mitochondrial ROS production in the denervated skeletal muscle cells.


Additional file 4: movie 3. Mitoflash recording of a denervated fiber in the presence of CsA; 004CsA72513_movie3. (AVI 976 kb)


Because CypD plays a critical role in controlling mPTP activity, we conducted immunoblot analysis to evaluate the protein expression level of CypD in both mitochondria and cytosol of the skeletal muscle following 24 h of denervation. We first confirmed the purity of mitochondrial and cytosol fractionation by using specific markers for mitochondria (COX-IV) and cytosol (GAPDH). As demonstrated in Additional file 5: Figure S2, the pure mitochondria fractions do not contain GAPDH, while the cytosol portion does not contain COX-IV, indicating no cross-contamination between the mitochondrial and cytosol fractionations. The level of CypD in mitochondria (CypD-mito) and cytosol (CypD-cyto) was quantified by normalization to the mitochondrial and cytosol specific markers, respectively, prior to the calculation of the CypD-mito to CypD-cyto ratio. We found that the ratio of CypD level in mitochondria to the CypD level in cytosol (CypD-mito/CypD-cyto) was significantly increased in the denervated muscle compared with the sham-treated muscle (sham 1.28 ± 0.20 vs Den 2.12 ± 0.31, *n* = 5, *p* < 0.05) (Fig. 1g and h). The data provide additional support that the denervation-induced mitoflash signal is likely related to CypD-dependent pore opening of the mPTP.

### Electric stimulation reduces mitoflash activity in both sham and denervated muscle fibers

**Fig. 2:**
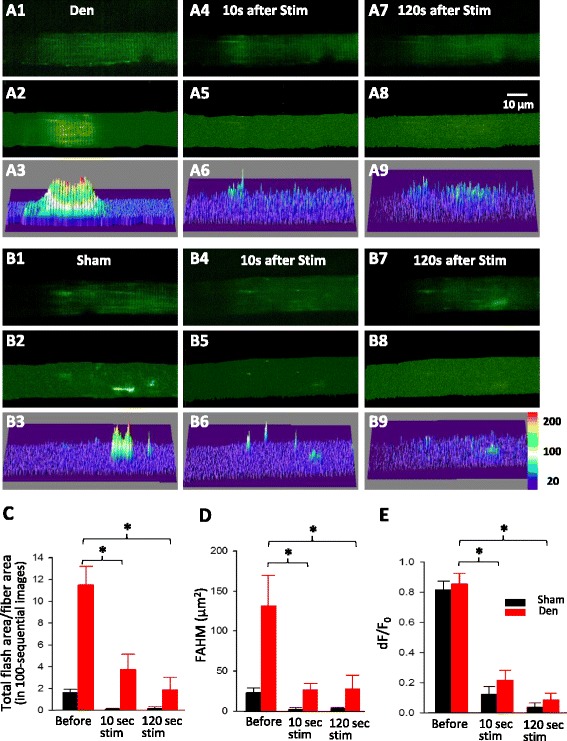
Electric stimulation eliminated the effects of denervation on the mitoflash activity. The representative confocal images of muscle fibers isolated from the denervated FDB muscle before field stimulation (Den) (**a1–3**), 10 s after the field stimulation (**a4–6**), and 120 s after the field stimulation (**a7–9**). Panels **a1**, **a4**, and **a7** show the initial fluorescence images at *t* = 0 s. Panels **a2**, **a5**, and **a8** show the peak intensity map of all mitoflashes detected in 100 recorded images. Panels **a3**, **a6**, and **a9** are the 3D surface plots of (**a2**, **a5**, and **a8**). The representative confocal images of muscle fibers isolated from the sham FDB muscle before field stimulation (sham) (**b1–3**), 10 s after the field stimulation (**b4–6**), and 120 s after the field stimulation (**b7–9**). Panels **b1**, **b4**, and **b7** show the initial fluorescence images at *t* = 0 s. Panels **b2**, **b5**, and **b8** show the peak intensity map of all mitoflashes detected in 100 recorded images. Panels **b3**, **b6**, and **b9** are the 3D surface plots of (**b2**, **b5**, and **b8**). The quantification of the mitoflashes are illustrated in **c** Total Flash Area/Fiber Area, **d** FAHM, and **e** the signal amplitude


The sciatic nerve transection isolates the muscle from the central nervous system and deprives it from any electric stimulation, which results in the absence of action potentials and physiological Ca^2+^ transients in the denervated muscle fibers. We hypothesize that the loss of physiological Ca^2+^ transients may contribute to the elevated mitochondrial ROS production. Thus, we tested whether restoration of physiological Ca^2+^ transient could eliminate the enhanced mitochondrial ROS product induced by denervation. We applied a brief field electric stimulation (350 ms) on isolated muscle fibers to initiate Ca^2+^ transients and evaluated the mitoflash activity shortly after the electric stimulation. Fibers that did not visually respond to stimulation were excluded from the study. Additional files 6 and 7 (movie 4 and 5) and Fig. 2 show representative images of muscle fibers isolated from the denervated (Fig. 2a1–9, Additional file 7: Movie 5) and sham muscles (Fig. 2b1–9, Additional file 6: Movie 4), before, 10 and 120 s after the electric stimulation. Quantitation of the mitoflash area (Total Flash Area/Fiber Area) of the muscle fiber (Fig. 2c) shows a persistent and significant decrease at 10 and 120 s respectively in both sham (sham 1.62 ± 0.46, 0.1 ± 0.09, 0.16 ± 0.22, *n* = 17–24; *p* < 0.001) and denervated (11.53 ± 2.87, 3.74 ± 2.38, 1.89 ± 1.87, *n* = 18–20; *p* < 0.001) muscle fibers. In addition, the flashing area in denervated fibers at 120 s after stimulation was reduced to a level similar to that observed in sham fibers before the stimulation (sham before stimulation, 1.62 ± 0.46, *n* = 24; Den + Stim at 120 s, 1.89 ± 1.87, *n* = 20 cells; *p* = 0.792). The changes were also observed in FAHM (Fig. 2d) in both sham (23.38 ± 9.33, 2.72 ± 1.92, 2.87 ± 3.45, *n* = 19–24; *p* < 0.001) and denervated (131.73 ± 65.79, 27.13 ± 13.26, 27.96 ± 29.62, *n* = 20 cells; *p* < 0.05) cells. Furthermore, the stimulation had a significant effect on the mitoflash amplitude dF/F_0_ (Fig. 2e) that was reduced at 10 and 120 s, respectively, in sham (0.82 ± 0.09, 0.12 ± 0.09, 0.04 ± 0.05, *n* = 19–24; *p* < 0.001) and denervation (0.86 ± 0.12, 0.22 ± 0.12, 0.09 ± 0.07, *n* = 20; *p* < 0.001). These results are in line with the effects of CsA on the mitoflash amplitude (Fig. 1f). However, compared with the effect of CsA, the data indicate that the electric stimulation has a more potent effect on reducing the mitoflash activity.

Additional file 6: Movie 4. Mitoflash recording of a denervated muscle fiber before electric stimulation; 005BeforeDenStim050614_movie4. (AVI 1634 kb)



Additional file 7: Movie 5. Mitoflash recording of a denervated muscle fiber 10 s after the electric stimulation; 005aDenStim050614_movie5. (AVI 1334 kb)


**Fig. 3:**
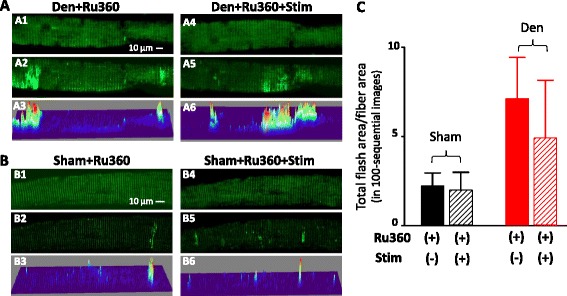
Ru360 eliminated the effect of field stimulation on mitoflash activity. The representative confocal images of muscle fibers isolated from the denervated FDB muscle fibers before field stimulation (Den **a1–3**), and 120 s after the field stimulation (**a4–6**) in the presence of 10 μM Ru360. Panels **a1** and **a4** show the initial fluorescence images at *t* = 0 s. Panels **a2** and **a5** show the peak intensity map of all mitoflashes detected in 100 recorded images. Panels **a3** and **a6** are the 3D surface plots of (**a2** and **a5**). The representative confocal images of muscle fibers isolated from the sham FDB muscle before the field stimulation (sham) (**b1–3**), 120 s after the field stimulation (**b4–6**). Panels **b1** and **b4** show the initial fluorescence images at *t* = 0 s. Panels **b2** and **b5** show the peak intensity map of all mitoflashes detected in 100 recorded images. Panels **b3** and **b6** are the 3D surface plots of (**b2** and **b5**) in the presence of 10 μM Ru360. The quantification of the Total Flash Area/Fiber Area of the mitoflashes is summarized in (**c**). Note that in the presence of Ru360, there are no significant changes in mitoflash activity before and after the field stimulation (*n* = 5, *p* > 0.05)

Since the mitoflash activity in the denervated muscle fibers was dramatically reduced by an electric field stimulation, we hypothesize that Ca^2+^ uptake into mitochondria may have a potential role in control of mitoflash activity during a physiological Ca^2+^ release transient. Thus, we tested whether pharmacological inhibition of the mitochondrial Ca^2+^ uptake by Ru360, an inhibitor of mitochondrial uniporter [19, 38] could block the effect of the field stimulation on the denervated muscle fibers. The control (sham) and denervated FDB muscle fibers were first incubated with 10 μM Ru360 for 30 min before receiving the field stimulation. As shown in Fig. 3, in the presence of Ru360, the field stimulation had no significant effect on the mitoflash activity of muscle fibers. The Total Flash Area/Fiber Area of denervated fibers in the presence of Ru360 before and after the stimulation are 7.12 ± 2.31 and 4.91 ± 3.24 (*n* = 5, *p* > 0.05). The (Total Flash Area/Fiber Area) of sham in the presence of Ru360 before and after stimulation is 2.22 ± 0.73 and 1.99 ± 0.99 (*n* = 5, *p* > 0.05). Similarly, FAHM of sham fibers before and after stimulation are 14.29 ± 3.96 and 9.26 ± 5.30 (*n* = 5, *p* > 0.05) and that of denervated fiber are 56.75 ± 15.89 and 61.20 ± 20.80, respectively, (*n* = 5, *p* > 0.05). The mitoflash intensity (dF/F_0_) before and after the field stimulation in the presence of Ru360 for sham fibers are 1.71 ± 0.13 and 1.71 ± 0.08 (*n* = 5, *p* > 0.05) and for denervated fibers are 1.71 ± 0.14 and 1.66 ± 0.09 (*n* = 5 *p* > 0.05). In addition, the application of Ru360 did not lead to significant changes in mitoflash activity in both sham and denervated muscle fibers before the electric stimulation (see Total Flash Area/Fiber Area in Figs. 1d, 2c, and 3c). These data suggest that the transient mitochondrial Ca^2+^ uptake is likely responsible for suppressing the mitoflash activity during a physiological Ca^2+^ release transient.

### Electrical stimulation and CsA treatment reduces the recurrence of mitoflash events in denervated skeletal muscle

**Fig. 4:**
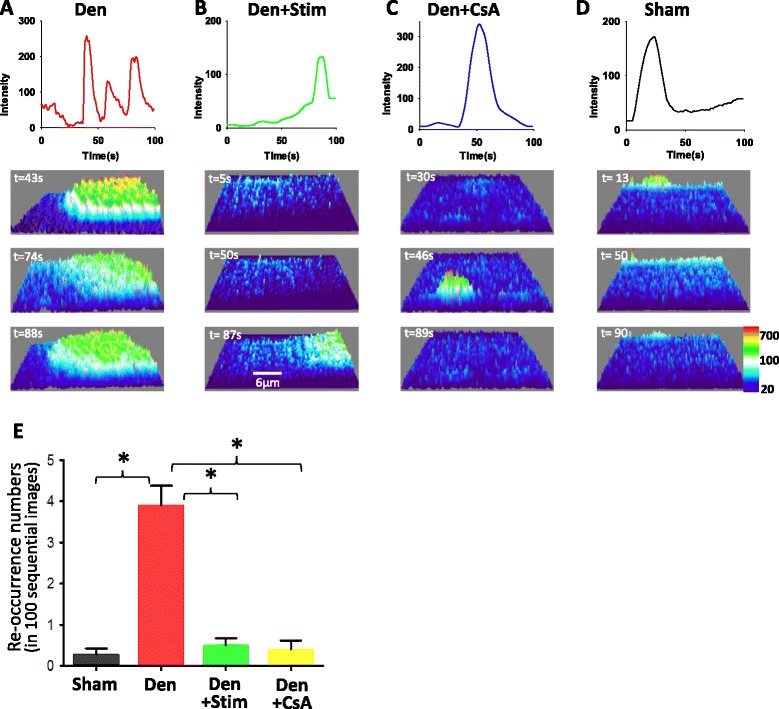
Repetitiveness of the mitoflash signal is reduced by field stimulation and CsA treatment. The temporal profile of an individual mitoflash patch at a local fiber region is illustrated for the conditions of denervation (Den) (**a**), denervation after 120 s of field stimulation (Den + Stim) (**b**), denervation plus CsA treatment (Den + CsA) (**c**), and the sham muscle fiber (**d**). Three representative 3D surface plots of the mitoflash patch for each condition are presented in the *lower panels*. **e** The summary *bar graph* for quantification of the re-occurrence numbers during the 100 sequential images (*n* = 10 for each group, *p < 0.01). Note that both field stimulation and CsA treatment reduced the repetitiveness of the mitoflash signal induced by the denervation

Two major changes of skeletal muscle fibers following denervation are the larger size of the mitoflash signal (FHAM) and the Flash Area/Fiber Area during the 100 s recording period (one image/s). The four- to fivefold increase in the Flash Area/Fiber Area in the denervated muscle fibers not only indicates more mitochondria being activated to generate mitoflash events but also suggests an increased frequency of mitochondria to produce mitoflash events during the 100 s recording time period. By focusing on a region of interest (ROI) when examining individual images recorded during the 100 s period, we found that mitochondria in the denervated muscle fibers displayed more recurrence of the mitoflash activity during the 100 s recording period. As shown in Fig. 4a, a denervated muscle fiber displayed mitoflash events three times during the 100 s recording period at one local fiber area (ROI), while in a sham muscle fiber, the mitoflash signal appeared only once during the 100 s recording period (Fig. 4d). The results further suggest that denervation indeed results in repetitive opening of the mPTP. Remarkably, both field stimulation and CsA treatment decreased the reoccurrence of the mitoflash signal induced by the denervation as shown in Fig. 4b–c. The reoccurrence numbers of mitoflash events during 100 sequential images for each experimental group are summarized in Fig. 4e.

### Mitochondria Ca^2+^ transient follows the physiological intracellular Ca^2+^ transient

**Fig. 5:**
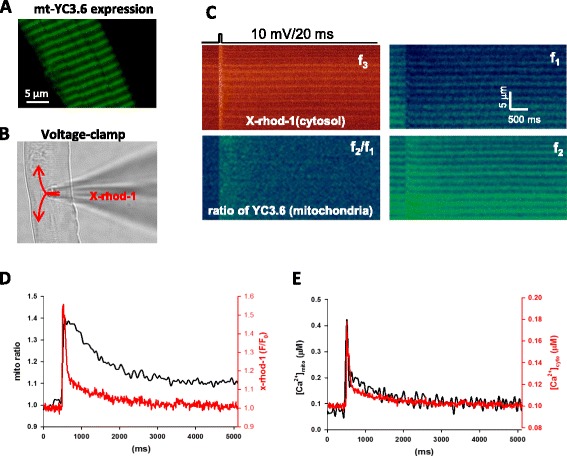
Simultaneous recording of cytosolic and mitochondrial Ca^2+^ transients. **a** A representative confocal image of FDB fiber expressing mt-YC3.6. Note the mitochondrial expression pattern of mt-YC3.6. **b** A representative image of an FDB fiber that is voltage-clamped through a glass pipette (from eight independent experiments). x-rhod-1 is delivered into the cytosol of the fiber through the pipette for recording of cytosolic Ca^2+^ transients. **c** Simultaneous recording of the dynamic changes of Ca^2+^ in both cytosol and mitochondria. *Panel f*_*3*_ is the fluorescence change of x-rhod-1 in cytosol during a brief pulse (20 ms). *Panels f*_*1*_ *and f*_*2*_ are the fluorescence changes of mt-YC3.6 at different emission wavelength. *Panel f*_*2*_ */f*_*1*_ is the ratio of mt-YC3.6 images. **d** The time course of cytosolic x-rhod-1 (*red trace*) and mitochondrial mt-YC3.6 ratio (*black trace*) following a brief depolarization pulse (20 ms). **e** The time course of quantified Ca^2+^ transients in cytosol (*red*) and mitochondria (*black*). Note the similar time courses of cytosolic and mitochondrial Ca^2+^ transients

The field electric stimulation of FDB fibers mimics the nerve stimulation by inducing action potentials in muscle fibers that lead to physiological Ca^2+^ release from the sarcoplasmic reticulum (SR) and dynamic changes of intracellular Ca^2+^ level named Ca^2+^ transients. We ask whether the free Ca^2+^ level inside mitochondria follows the intracellular Ca^2+^ transient and whether it may be associated with mitochondrial ROS production. We have previously established a way to quantify mitochondrial Ca^2+^ uptake in skeletal muscle fibers using mitochondria-targeted Ca^2+^ biosensor mt-YC3.6 under the voltage-clamp condition and demonstrated that the level of mitochondria Ca^2+^ synchronizes with the cytosolic Ca^2+^ transient during a brief membrane depolarization with 10 ms duration [20]. Since the duration of the field stimulation applied to reduce the denervation-induced mitoflash signal was 350 ms, we examined whether Ca^2+^ transients in mitochondria also follows the time course of the cytosolic Ca^2+^ transients during a prolonged membrane depolarization using FDB muscle transfected with mt-YC3.6. Ideally, if we could measure the mitochondrial Ca^2+^ level in cpYFP muscle fibers, the fluorescence spectra of cpYFP and mt-YC3.6 overlap each other. Thus, we have quantified mitochondrial Ca^2+^ uptake following a voltage-induced Ca^2+^ release in mt-YC3.6 muscle fibers without cpYFP expression. As shown in Fig. 5a, the muscle fiber transfected with mt-YC3.6 has mitochondria-targeted pattern. The YC3.6 protein functions as a ratiometric Ca^2+^ probe [39] because its fluorescence changes in opposite directions at two wavelengths (*f*_1_ and *f*_2_) as shown in Fig. 5c in response to Ca^2+^ release from the SR (Fig. 5c, *f*_3_). *f*_2_ vs *f*_1_ wavelengths represent the Ca^2+^ bound vs unbound states, respectively. The ratio of *f*_2_/*f*_1_ (mito ratio) indicates an increase of [Ca^2+^] inside mitochondria upon voltage-induced Ca^2+^ release (Fig. 5d, black trace). The dynamic change of free Ca^2+^ level in mitochondria (mitochondrial Ca^2+^ transient) (Fig. 5e, black trace) was calculated using the method established in our previous work [20].

The patch pipette solution contained x-rhod-1, a fast cytosolic Ca^2+^ indicator with excitation and emission spectra distinct from those of mt11-YC3.6. The x-rhod-1 delivered into the cytosol of the muscle fiber (Fig. 5b) allows the recording of Ca^2+^ transients in the cytosol simultaneously with mitochondrial signal recording of mt-YC3.6 (Fig. 5c, *f*_3_). The change in fluorescence intensity *F*/*F*_0_ (*t*) of x-rhod-1 (Fig. 5d, red trace) was used to derive the intracellular Ca^2+^ transient, and the changes in mito ratio *f*_3_ (*f*_2_/*f*_1_) (Fig. 5d, black trace) was used to derive the mitochondrial Ca^2+^ uptake using the same method described in our previous work [20] (Fig. 5e). Figure 5e demonstrates that the dynamic change of Ca^2+^ level in mitochondria (Fig. 5e, black trace) indeed follows the dynamic change of Ca^2+^ level in the cytosol (Fig. 5e, red trace). Following a short (20 ms) depolarization stimulation, the time courses of Ca^2+^ transients are very similar in both mitochondria and cytosol.

**Fig. 6:**
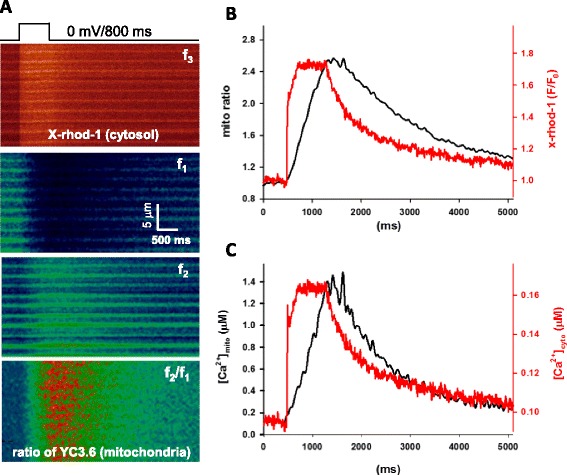
Mitochondrial Ca^2+^ transient synchronized the time course of the cytosolic Ca^2+^ transient during an elongated depolarization pulse. **a** The representative confocal images of cytosolic x-rhod-1 and mitochondrial mt-YC3.6 during this 800 ms depolarization (from eight independent experiments). **b** The time course of cytosolic x-rhod-1 (*red trace*) and mt-YC3.6 ratio (*black trace*) during a 800 ms depolarization pulse. **c** The time course of quantified Ca^2+^ transients in cytosol (*red*) and mitochondria (*black*). Although the mitochondrial Ca^2+^ transient shows a delay to reach the peak value, the time course of mitochondrial Ca^2+^ transient did synchronize with the cytosolic Ca^2+^ transient

We next examined whether mitochondrial Ca^2+^ transients synchronized with the cytosolic Ca^2+^ transients during a longer depolarization pulse of 800 ms. As demonstrated in Fig. 6, the FDB muscle fiber expressing mt-YC3.6 was stimulated with a depolarization pulse of 800 ms. This elongated stimulation induced an elongated Ca^2+^ release signal in the cytosol (Fig. 6a, the panel *f*_3_ and Fig. 6b, the red trace of x-rhod-1). The simultaneously recorded mt-YC3.6 signals (Fig. 6a, panels *f*_1_ and *f*_2_) provided a recording of mitochondrial Ca^2+^ uptake represented by the ratio of *f*_2_/*f*_1_ (Fig. 6a, panel *f*_2_/*f*_1_). Both quantified cytosolic and mitochondrial Ca^2+^ transients induced by the elongated depolarization pulse are depicted in Fig. 6c (the red trace for cytosolic [Ca^2+^] and the black trace for mitochondrial [Ca^2+^]). During the elongated depolarization, there was a delay for mitochondrial Ca^2+^ transient to reach its peak value. However, the mitochondrial Ca^2+^ transient was synchronized with the cytosolic Ca^2+^ transient. The data demonstrates that the Ca^2+^ level in mitochondria indeed follows the dynamic changes of the Ca^2+^ level in cytosol. In the case of denervation, no action potential is initiated by the motor neurons, thus there are no cytosolic Ca^2+^ transients, and therefore, no mitochondrial Ca^2+^ transients either. It is possible that dynamic alterations of mitochondrial Ca^2+^ transients may be essential to maintain the normal mitochondrial function in skeletal muscle. The absence of dynamic alterations of mitochondrial Ca^2+^ transients may be associated with the enhanced mitoflash activity, which is linked to the enhanced mitochondrial ROS generation following the denervation.

### Using MitoSOX Red to evaluate the role of intracellular Ca^2+^ transients on mitochondrial ROS production

**Fig. 7:**
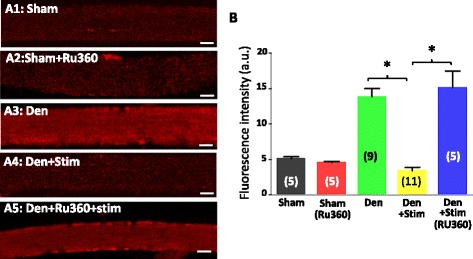
The effect of denervation on mitochondrial ROS production evaluated by MitoSOX Red. Muscle fibers were loaded with the mitochondrial superoxide indicator, MitoSOX Red. Panels **a1** and **a2** are the representative images of sham muscle fibers in the absence and presence of 10 μM Ru360, which inhibits the mitochondrial Ca^2+^ uptake through the mitochondrial uniporter. Note that Ru360 has no significant effect on mitochondrial superoxide production level. Panel **a3** is the representative image of denervated muscle fibers. Panels **a4** and **a5** are the representative images of denervated muscle fibers received the electric field stimulation in the absence and presence of 10 μM Ru360. b Quantification of the relative MitoSOX Red fluorescence intensity at those different experimental conditions. Note that the denervated muscle fibers show enhanced MitoSOX Red fluorescence intensity (**a3**), while the electric field stimulation reduced the denervation-induced increase in MitoSOX Red intensity (**a4**), and Ru360 significantly blocked the effect of the field stimulation on the denervated muscle fiber (**a5**) (*n* = 5–11, *p < 0.05). *Bar*, 20 μm

Because of the ongoing debate of the mt-cpYFP sensitivity on ROS and pH [34], we used MitoSOX Red, a commercially available dye to further evaluate the response of denervated muscle fibers to the electric field stimulation. MitoSOX Red is a fluorogenic dye for highly selective detection of superoxide and is rapidly and selectively targeted to mitochondria. Once in mitochondria, MitoSOX Red is oxidized by superoxide and exhibits red fluorescence (M36008, Invitrogen). This reaction is not reversible and MitoSOX Red is not able to follow the dynamic changes of the mitochondrial superoxide level before and after the field stimulation in a single muscle fiber. However, its relative fluorescent intensity can be used to compare the basal mitochondrial superoxide production level among different muscle fibers when experimental conditions, such as the dye loading and the confocal microscope setting, are kept the same. We first loaded the FDB muscle fibers derived from the sham and the denervated legs of the same mouse with MitoSOX Red. As demonstrated in Figs. 7a1–2 and 6b, the fluorescent intensity of MitoSOX Red is significantly higher in the denervated muscle fibers (sham 6.29 ± 2.21 vs denervation 13.87 ± 3.80, *n* = 5, 11, *p* < 0.001). In the second set of experiments, we tested whether the electric field stimulation could reduce the fluorescent intensity of MitoSOX Red in the denervated FDB muscle fibers. The denervated FDB muscle fibers were first set to a glass bottom culture dish. A few denervated muscle fibers were selected to receive the electric field stimulation. Immediately after the electric field stimulation, the MitoSOX Red was added into the dish following the same dye loading procedure. The MitoSOX Red intensity of the denervated muscle fibers was then evaluated. As shown in Figs. 7a3 and 6b, the denervated muscle fibers received the field stimulation showed significantly lower MitoSOX Red intensity (3.41 ± 1.41, *n* = 9, *p* < 0.001 compared to the fibers without field stimulation). This set of data suggest that mitochondria in denervated skeletal muscle indeed response to the physiological intracellular Ca^2+^ transients with reduced superoxide production.

Since our voltage-clamp data demonstrated that Ca^2+^ level in mitochondria followed the dynamic changes of cytosolic Ca^2+^ level and Ru360 blocked the effect of electric stimulation on mitoflash activity (Fig. 3), we hypothesize that mitochondrial Ca^2+^ uptake may affect the superoxide production during a physiological Ca^2+^ release transient. As in Fig. 3, we further tested whether pharmacological inhibition of the mitochondrial Ca^2+^ uptake by Ru360 could block the effect of the field stimulation on the denervated muscle fibers labeled with MitoSOX Red. The denervated FDB muscle fibers were first incubated with 10 μM Ru360 for 30 min before receiving the field stimulation. Immediately, following the field stimulation (still in the presence of 10 μM Ru360), the denervated FDB fibers were loaded with MitoSOX Red for evaluation of mitochondrial superoxide level. As shown in Fig. 7a4 and b, in the presence of Ru360, the field stimulation no longer reversed the elevated mitochondrial superoxide level in the denervated muscle fibers (15.15 ± 5.16, *n* = 5, *p* < 0.05 compared to the denervated fibers without Ru360), suggesting that the transient mitochondrial Ca^2+^ uptake is likely responsible for suppressing the mitochondrial ROS production during a physiological Ca^2+^ release transient. We also conducted a control experiment to test if Ru360 has a potential effect on mitochondrial ROS production. As demonstrated in Fig. 7a1 and a2, Ru360 has no significant influence on mitochondrial superoxide production level.

## Discussion

Previously published studies on how Ca^2+^ signaling regulate mitochondrial ROS production in skeletal muscle mainly focused on the effect of a steady state level of Ca^2+^ inside mitochondria [40]. Here, we provide the first evidence that the dynamic uptake of Ca^2+^ by mitochondria may also be a key player in maintaining the physiological function of mitochondria. We showed that the time course of free Ca^2+^ level inside mitochondria follows the time course of cytosolic Ca^2+^ transient and that physiological changes in mitochondrial Ca^2+^ levels could be essential for maintaining the functional integrity of mitochondria. Following denervation, there is no action potential initiated in muscle fibers, and therefore, no Ca^2+^ transients in the cytosol and mitochondria. Mitochondria respond to this condition with increased ROS production that is associated with mPTP opening. Restoring the physiological Ca^2+^ transients by electric stimulation reduced the mitochondrial ROS production and stopped the repetitive mPTP opening in the denervated muscle fibers.

Motor neuron innervation is critical for the growth and maintenance of muscle fibers. Denervation is known to cause muscle atrophy, manifested in age-dependent sarcopenia and other neurological diseases, such as amyotrophic lateral sclerosis (ALS). Studies by other investigators have shown that denervation leads to compromised EC coupling machinery in skeletal muscle [41] and altered mitochondrial metabolic function [42, 43]. The increase in ROS generation is a common event in skeletal muscle mitochondria under a variety of pathological conditions associated with denervation-induced muscle atrophy [10]. Excessive ROS generation contributes to apoptotic or necrotic cell death [22]. Biochemical assay has revealed that the ROS generation in muscle mitochondria was dramatically increased after several days of surgical sciatic nerve transection [10, 44]. It is believed that enhanced ROS generation is a common factor in the mechanism underlying denervation-induced muscle atrophy and the related downstream signaling pathways have been extensively studied [2, 11]. However, the initial signal that causes changes in the mitochondrial network and ROS production of skeletal muscle in response to denervation remains unknown. It is not clear whether the dynamic changes of the Ca^2+^ level in the cytosol and mitochondria are implicated in this process. Here, we provide evidence that physiological Ca^2+^ transient is required to maintain the integrity of mitochondrial function in the physiological condition. Absence of the physiological Ca^2+^ transients is likely a direct cause of enhanced mitochondrial ROS production that is associated with the repetitive opening of the mitochondrial mPTP.

Wang et al. developed a mitochondrial-targeted, circularly-permutated yellow fluorescent protein (mt-cpYFP) as an indicator of ROS production and energy metabolism of individual mitochondrion in live cells including skeletal muscle cells [25–27]. Their studies revealed that ROS is produced in transient waves at the level of individual mitochondria, a phenomenon known as the mitoflash [25]. Using the same transgene, we produced the mt-cpYFP transgenic mice in our laboratory and investigated the effect of denervation on mitochondrial function by monitoring the dynamic changes of mitoflash signals in mitochondria of live muscle fibers. Twenty-four hours following the sciatic nerve transection, denervated muscle fibers show an excessive increase in the mitoflash signal, specifically in the signal size (FAHM) that was four times larger than in control fibers. The data indicate that in a single muscle fiber, more mitochondria are activated to produce mitoflash signals in response to denervation. In addition, the Total Flash Area/Fiber Area of the mitoflash signal recorded during the 100-sequential-imaging time period was four times more than the controls, indicating a higher frequency of individual mitochondria to produce mitoflash activity.

In both skeletal and cardiac muscle cells, mitochondria form a structural and functional network [30, 45, 46]. Imaging of mitoflash signal of skeletal muscle in mt-cpYFP mice has also revealed functional mitochondrial network [27]. In the current study, the dramatic increase in the size of the mitoflash signal (FAHM) could indicate that the mitochondrial network is physically more connected following denervation. However, this is unlikely, as a study by Romanello et al. has reported that denervation promotes mitochondrial fission activity, which should reduce the physical connection between mitochondria [47]. Our previous study has also identified a reduced mitochondrial network with enhanced fission activity in skeletal muscle of an ALS mouse model (G93A), in which skeletal muscle experiences denervation during ALS progression [30]. As identified in cardiac myocytes, the release of ROS from a subset of mitochondria can trigger ROS release from the adjacent mitochondria [48–50]. The dramatically enhanced size of mitoflash signal in skeletal muscle is likely an indication of the ROS-induced ROS release in mitochondria that is augmented by denervation. Other kinetic parameters such as the signal amplitude and FDHM stay the same, indicating that mitoflash kinetics was not affected by a short period (24 h) of denervation.

While mitochondria take up Ca^2+^ during skeletal muscle contraction [19, 20, 51], the mitochondrial Ca^2+^ uptake seems to be a double-edged sword for the fate of cells [22, 40]. Under normal conditions, mitochondrial Ca^2+^ uptake is a physiological stimulus for ATP synthesis [21, 52, 53]. The elevated mitochondrial Ca^2+^ level leads to a coordinated upregulation of oxidative phosphorylation machinery, resulting in higher mitochondrial ATP output to meet the energy demanding of the cells [54, 55]. This is required to meet the increased contractile force during muscle contraction. In addition, the mouse model (MCU^−/−^) with global knockout of mitochondrial calcium uniporter (MCU) showed a smaller body size and impaired skeletal muscle performance along with absence of mitochondrial Ca^2+^ uptake in isolated skeletal muscle mitochondria, indicating that mitochondrial Ca^2+^ uptake plays an important role in skeletal muscle development and performance [56]. However, Ca^2+^ overload in mitochondria is also a pathological stimulus of ROS generation [22]. It has been shown that prolonged skeletal muscle inactivity (such as muscle disuse) leads to an increased resting cytosolic free Ca^2+^ level, that in turn overloads mitochondria to stimulate the ROS production [23, 24]. While previous published studies on how Ca^2+^ signaling regulate mitochondrial ROS production in skeletal muscle mainly focused on the effect of a steady state level of Ca^2+^ inside mitochondria, it is not known whether the dynamic Ca^2+^ uptake by mitochondria has a role in maintaining the mitochondrial functional integrity. Recording of the mitoflash signal in a skeletal muscle fiber following a short period of denervation allowed us to discover the early response of mitochondria to the absence of physiological cytosolic Ca^2+^ transients, which limits mitochondrial Ca^2+^ uptake and leads to an excessive increase in mitochondrial ROS production. Our result is in line with the study by Csordas et al. [57], in which they found that destruction of the ER-mitochondria linkage causes the specific loss of the IP_3_R-mediated Ca^2+^ transfer to mitochondria that stimulates oxidative metabolism in a cultured mammalian cell line [57]. Most importantly, we demonstrated that the early increase of mitochondrial ROS production was immediately diminished by restoring a train of physiological intracellular Ca^2+^ transients in denervated muscle fibers and that the transient mitochondrial Ca^2+^ uptake could be a key regulator of mitochondrial mPTP activity. Interestingly, recent studies from other research groups also provided evidence of MCU-dependent mitochondrial Ca^2+^ uptake in protecting denervation-induced skeletal muscle atrophy by using virus-mediated overexpression of MCU [58, 59]. While it is very well known that Ca^2+^ overload into mitochondria leads to mPTP opening and ROS production, one of the major findings in the present study is that the denervation-induced ROS elevation could be reduced by electrical stimulation, indicating an unexpected role of the Ca^2+^ uptake into mitochondria for control of ROS production. While a consistently elevated intracellular Ca^2+^ level leads to the Ca^2+^ overload in mitochondria and mPTP opening, the absence of physiological Ca^2+^ transients following denervation, which leads to the absence of the dynamic Ca^2+^ uptake into mitochondria, also triggers mPTP opening and initiates mitochondrial dysfunction. We speculate that there may be a biphasic dependence of mPTP on Ca^2+^ level or direct response to SR Ca^2+^ release. The molecular composition of mPTP is still incompletely understood. It is unclear what are the molecular mechanisms underlying the different responses of mitochondria to a consistently elevated intracellular Ca^2+^ level and to the absence of physiological Ca^2+^ transients following denervation. Future studies are needed to further understand the molecular mechanism underlying the initial response of mitochondria to denervation in skeletal muscle.

While denervation eliminates neuromuscular transmission and myoplasmic Ca^2+^ transients, other muscle adaptations and changes can occur during the first 24 h after denervation. One possibility is that mitochondrial and/or cellular ROS detoxification mechanisms (e.g., SOD, GSH reductase, catalase, and thioredoxin) may be reduced after denervation, and thus, allow for aberrant cell-wide propagation of otherwise spatially restricted mitochondrial ROS-related events. If bursts of ROS-related mitoflash activity are initiated by low-level increases in ROS, then a breakdown of cellular ROS detoxification mechanisms following denervation could also have a contribution to the observed increase in cell-wide waves of ROS-induced ROS release. Thus, changes to denervated fibers in addition to the loss of cytoplasmic Ca^2+^ transients and mitochondrial Ca^2+^ uptake are needed to explain the observed enhancement in mitoflash activity. More studies are needed to further understand the molecular mechanism for early response of skeletal muscle to denervation.

One concern about our result is the possibility that the drastic reduction of the mitoflash signal in the denervated muscle fibers following the tetanic field stimulation is due to the cell damage caused by the tetanic field stimulation. We were aware of the study of mitoflash signal on normal skeletal muscle by Wei and colleagues who reported that the mitoflash frequency was significantly increased following a 2-s tetanic stimulation and markedly decreased following prolonged 20-s tetanic stimulation [37]. As a result, the rationale for our experiment design is to avoid the 2-sec tetanic stimulation that already showed promoting mitoflash activity. We also decided not to use 20-s stimulation, as we do not know if the 20-s tetanic stimulation could cause artificial responses in our study. Instead, we applied a tetanic stimulation with much shorter duration (350 ms). Thus, the reduction in mitoflash signal observed in the denervated muscle fibers following such a brief electrical stimulation is unlikely due to the physical damage caused by the field stimulation. Interestingly, this brief stimulation also suppressed the mitoflash activity in the sham muscle. Our data together with the previous study by Wei et al., suggest that the response of mitochondria to tetanic stimulation is dynamic, and the underlying molecular mechanisms may be different between the 350-ms stimulation and 2-s stimulation. It is not a surprise to see that a sevenfold longer electric stimulation (2 s) enhances the mitoflash activity, as it likely promotes more mitochondrial Ca^2+^ uptake, which is known to promote the function of the respiratory chain reaction.

We previously demonstrated that mitochondria in skeletal muscle take up Ca^2+^ following a brief electric stimulation [20]. Here, we showed that during prolonged membrane depolarization, mitochondrial Ca^2+^ transient also follows a similar time course as the cytosolic Ca^2+^ transient in skeletal muscle fibers. Our results support the hypothesis that the dynamic levels of Ca^2+^ inside mitochondria follow the time course of the cytosolic Ca^2+^ transients initiated by motor neuron activation in the physiological condition and that the dynamic change in mitochondrial Ca^2+^ level could serve as a sensor to decode the nerve innervation. While Ca^2+^ overload in mitochondria triggers more ROS production [22], our results indicate that absence of the physiological mitochondrial Ca^2+^ uptake also play a role for maintaining the integrity of normal mitochondrial function.

The coupling of mitoflash and mPTP opening has been well characterized in both cardiac and skeletal muscle [27, 35]. It has been shown that mt-cpYFP fluorescence is transiently increased when the mitochondrial membrane potential (measured by TMRE) is depolarized during a dual recording, indicating a dynamic coupling of the mitoflash event and the mPTP opening. Several lines of evidence have suggested a strong connection between CypD-related mPTP opening and the mitoflash signal in cardiac muscle but not in normal skeletal muscle. Indeed, manipulation of mPTP had a major impact on the mitoflash signal in cardiac myocytes. For example, activators of mPTP, like atractyloside, increased the mitoflash frequency in cardiac myocytes [26] but not in skeletal muscle fibers [36]. In contrast, CypD inhibitors such as cyclosporine A (CsA) caused a reduction in the mitoflash amplitude and kinetics in cardiac myocytes [26]. However, CsA did not appear to affect the mitoflash signal in skeletal muscle fibers under normal physiological conditions [36, 37], indicating that the mitoflash signal in normal skeletal muscle may not be related to the activity of CypD at physiological conditions. Remarkably, we demonstrated that in the case of denervation, the enhanced mitoflash signal might directly associated with the CypD-related opening of mPTP in skeletal muscle fibers. Incubation with 1 μM CsA for 30 min significantly reduced the Total Flash Area/Fiber Area, FAHM, and the amplitude of the mitoflash in denervated muscle fibers. CsA inhibits mPTP opening by binding to Cyclophilin D (CypD) [60]. Thus, the apparent relationship between mPTP and the mitoflash signal seems to be related on CypD in the denervated muscle. It has been shown that knocking down CypD with siRNA, a major component related to mPTP, significantly reduced mitoflash frequency in cardiac myocytes [26, 61]. The opposite occurred upon CypD overexpression [61]. Based on a biochemical study, Csukly K et al. has shown that following 3 weeks of denervation, the isolated mitochondria from denervated muscle have enhanced vulnerability to Ca^2+^-induced mPTP opening, and this Ca^2+^-induced mPTP opening can be blocked by the application of CsA. In addition, the relative level of CypD is increased in mitochondrial fraction of the denervated skeletal muscle, indicating a CypD-related mPTP opening in denervated muscle [42]. In line with this discovery, we found that 24-h denervation led to an increase in the ratio of CypD-mito/CypD-cyto. The increased ratio of CypD-mito/CypD-cyto could reflect increased degradation of the cytosolic CypD, increased stability of mitochondrial CypD, or increased translocation of CypD from the cytosol to mitochondria. Nevertheless, the increased ratio indicates a relative change of CypD in mitochondria fraction. This result together with the pharmacological study of CsA suggests that CypD is likely involved in regulating mPTP activity and mitochondrial ROS production in skeletal muscle following 1 day of denervation. Although it is very well established that CsA inhibits mPTP by binding to CypD and has been extensively used as an investigative tool for mPTP research, it is also known that CsA inhibits calcineurin, a calcium dependent dephosphorylation enzyme [60, 62]. Studies by Cereghetti et al. and Cribbs and Strack demonstrate the role of calcineurin in dephosphorylation of Drp1, which promotes mitochondrial fission [63, 64]. It has been found that CsA promotes mitochondrial fusion through inhibition of calcineurin [63, 65]. Thus, it is possible that the reduced mitoflash activity in the presence of CsA may also partially due to the enhanced mitochondrial fusion, which indirectly affects the mitochondrial ROS production. Nevertheless, our result with relatively enhanced mitochondrial CypD level and the published study showing an enhanced CypD expression level in skeletal muscle with prolonged denervation suggest a potential involvement of the CypD-related mPTP opening in skeletal muscle in response to denervation. The CypD-related mPTP opening is likely an early event in skeletal muscle mitochondria in response to denervation. Most importantly, for the first time, our data demonstrated that the physiological Ca^2+^ transients and the following mitochondrial Ca^2+^ uptake play a key role in maintaining the functional integrity of mitochondria through a potential mechanism by regulating the mPTP-related mitochondrial ROS production.

Although mt-cpYFP has been characterized and used as a biomarker of mitochondrial ROS generation [26, 36, 37], there are concerns about the specificity of the mitoflash signal. It has been suggested that mitoflash may also report ATP [66] or pH changes inside mitochondrial matrix [34, 67]. Nevertheless, the mitoflash signal may reflect the status of the ROS-related metabolic function of mitochondria, and cpYFP likely can report both ROS and pH signal of mitochondria, and is thus a robust biosensor for mitochondrial function [68, 69]. However, the portion of ROS vs pH signal reported by the mitoflash signal could be context-dependent [35]. Such detailed quantification will require additional measurement, which is not the focus of the current study. A recent study by Ding et al. indicates that mitoflash represents a dynamic signal of mitochondrial ROS production and energy metabolism [25]. In our current study, mitoflash signal served as a unique biosensor that allowed us to dissect the early response of mitochondria to denervation and to identify the role of physiological Ca^2+^ transients in maintaining the functional integrity of mitochondria in skeletal muscle. Due to the ongoing debate of mt-cpYFP sensitivity on ROS and pH [34], we used MitoSOX Red, a separate fluorescent indicator for mitochondrial superoxide detection, to further evaluate the response of denervated muscle fibers to electrical stimulation. Although MitoSOX Red is not able to detect the dynamic changes of mitochondrial ROS production, its fluorescence intensity can report the basal level of mitochondrial superoxide production. Our MitoSOX Red data confirmed that mitochondrial ROS production was enhanced following 24 h of denervation and that electrical stimulation could reverse mitochondrial ROS production to basal level.

## Conclusions

In summary, we have taken advantage of the transgenic mt-cpYFP mice that allowed us to evaluate the early dynamic response of skeletal muscle to denervation at the mitochondrial level of live muscle fibers. For the first time, our study reveals that physiological Ca^2+^ transient is important for maintaining the normal mitochondrial metabolic function by regulating the mPTP-associated mitochondrial ROS generation. Combining voltage-clamp technique and live skeletal muscle cell imaging study, we showed that the Ca^2+^ level inside mitochondria of skeletal muscle is synchronized with the dynamic change of cytosolic Ca^2+^ level under physiological condition. In the case of denervation, there are no Ca^2+^ transients initiated in the cytosol and thus no dynamic alterations in mitochondrial Ca^2+^ level. Our study suggests that the dynamic change of Ca^2+^ level inside mitochondria regulates the mitochondrial ROS generation as a response to the cytosolic Ca^2+^ transient in the physiological condition. Additionally, mitochondria in denervated muscle fibers could sense the absence of the physiological Ca^2+^ transients and respond with enhanced ROS generation that could initiate downstream signaling toward mitochondrial dysfunction. Future studies should focus on the detailed molecular basis underlying the mitochondrial response to the absence of physiological Ca^2+^ transients.

## Additional files

Additional file 1: Figure S1. Live cell imaging shows the mitochondrial targeting of mt-cpYFP in skeletal muscle of the transgenic mice (cpYFP). mt-cpYFP targeted well to mitochondria in skeletal muscle of the transgenic mice cpYFP. The representative images of FDB muscle fibers enzyme-isolated from the transgenic mouse cpYFP were incubated with 50 nM TMRE for 10 min. A. Muscle fibers expressing mt-cpYFP. B. TMRE marked mitochondria of the same muscle fibers in A. C. Overlay of A and B indicates the targeting of mt-cpYFP to mitochondria. (PDF 304 kb)

Additional file 5: Figure S2. The pure mitochondria and cytosol fractions do not show contamination from each other. The COX-IV antibody only detects bands in pure mitochondrial fraction, not in cytosol fraction, while the GAPDH antibody only detects bands in the cytosol fraction. (PDF 174 kb)

## Abbreviations

ALS: Amyotrophic lateral sclerosis
CsA: Cyclosporine A
CypD: Cyclophilin D
E-C coupling: Excitation-contraction coupling
FAHM: Full amplitude of half maximum
FDB: Flexor digitorum brevis
FDHM: Full duration of half maximum
mPTP: Mitochondrial transition pore
mt-cpYFP: Mitochondrial targeted biosensor
ROS: Reactive oxygen species
SR: Sarcoplasmic reticulum
