# Supplementary figures and images for: Absence of physiological Ca2+ transients is an initial trigger for mitochondrial dysfunction in skeletal muscle following denervation
Source: Skelet Muscle. 2017 Apr 10;7:6. doi: 10.1186/s13395-017-0123-0 (PMC5387329; doi:10.1186/s13395-017-0123-0)

Figure S1

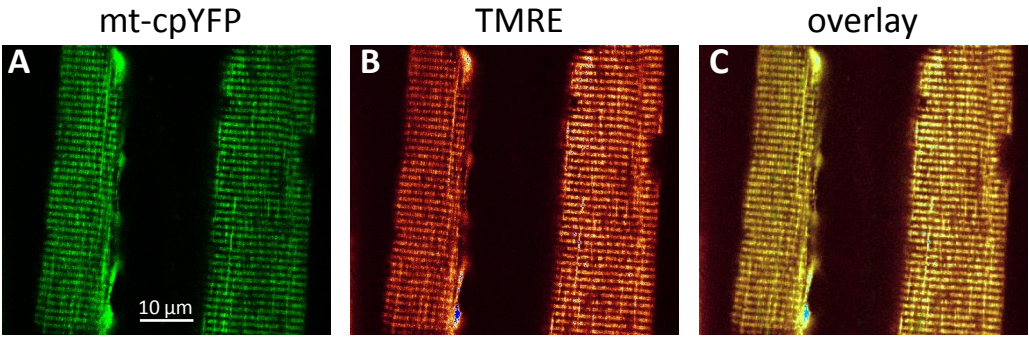

Supplement: Supplementary file 6 — Live cell imaging shows the mitochondrial targeting of mt-cpYFP in skeletal muscle of the transgenic mice (cpYFP). mt-cpYFP targeted well to mitochondria in skeletal muscle of the transgenic mice cpYFP. The representative images of FDB muscle fibers enzyme-isolated from the transgenic mouse cpYFP were incubated with 50 nM TMRE for 10 min. A. Muscle fibers expressing mt-cpYFP. B. TMRE marked mitochondria of the same muscle fibers in A. C. Overlay of A and B indicates the targeting of mt-cpYFP to mitochondria. (PDF 304 kb) [file 13395_2017_123_MOESM1_ESM.pdf]

Figure S2

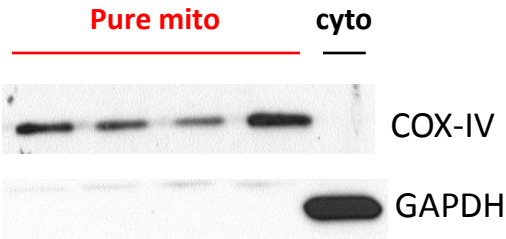

Supplement: Supplementary file 7 — The pure mitochondria and cytosol fractions do not show contamination from each other. The COX-IV antibody only detects bands in pure mitochondrial fraction, not in cytosol fraction, while the GAPDH antibody only detects bands in the cytosol fraction. (PDF 174 kb) [file 13395_2017_123_MOESM5_ESM.pdf]
